# Feasibility Study of Bariatric Surgery in a Rat Model of Spinal Cord Injury to Achieve Beneficial Body Weight Outcome

**DOI:** 10.1089/neur.2022.0027

**Published:** 2022-08-08

**Authors:** Gregory M. Holmes, Lisa B. Willing, Nelli Horvath, Andras Hajnal

**Affiliations:** Department of Neural and Behavioral Sciences, Penn State University College of Medicine, Hershey, Pennsylvania, USA.

**Keywords:** bariatric surgery, obesity, spinal cord injury, vertical sleeve gastrectomy, weight loss surgery

## Abstract

Approximately two thirds of spinal cord injury (SCI) persons become overweight or obese. Obesity increases the risk of developing type 2 diabetes and limits self-help techniques. Weight-loss surgery (WLS), including vertical sleeve gastrectomy (VSG), is regarded as highly effective in the long-term treatment of obesity and remission of associated type 2 diabetes. Given the increased risk of obesity post-SCI, WLS offers an attractive intervention strategy. Alterations in the physiology of energy homeostasis after SCI necessitate that SCI persons should not be regarded as similar to able-bodied persons. Because of current knowledge gaps, it is unknown whether an obese phenotype with SCI will respond to WLS similarly to the neurally intact obese phenotype. Therefore, this study tested the hypothesis that the VSG procedure is well tolerated and effective in an animal model of high-thoracic (T3) SCI. In Wistar male rats, subsequent to a 2-week recovery period after T3-SCI, but not control laminectomy surgery, daily consumption of a high-fat diet (HFD; 60% kcal from fat) was elevated over 4 weeks preceding VSG. After a 2-week recovery period post-VSG, HFD consumption in T3-SCI rats over a 4-week monitoring period returned to levels comparable to control. Body weight was significantly reduced in T3-SCI rats and remained reduced whereas control rats regained body weight. Further, no adverse complications directly attributable to the VSG procedure were identified. Thus, this rodent model is a viable tool for addressing fundamental questions regarding the mechanisms leading to obesity post-SCI and the development of translational strategies.

## Introduction

The profound effects of spinal cord injury (SCI) are widely recognized by clinical and pre-clinical scientists whereby derangements of somatic and autonomic neural control subsequent to the injury are accompanied by a reduction in physical activity. Ultimately, afflicted persons experience dysregulation of energy homeostasis that very quickly progresses to obesity (reviewed in a previous work^1^). For the able bodied, obesity is defined by a body mass index (BMI) >30 kg/m^2^. The utility of the BMI for the SCI population is debatable and may well underestimate the percent of fat mass after SCI.^1,2^ Further, as a result of the disproportionate ratio of fat to lean tissue attributable to sarcopenia, more persons with SCI are prone to the same constellation of unhealthy sequelae as obese persons, including glucose dysregulation, insulin resistance, and cardiovascular disease.^3,4^ These aggregate comorbidities are often referred to as metabolic syndrome^5^ and display a higher prevalence in the SCI population compared to the general population.^6–8^

Interventions for weight management involving lifestyle changes are often ineffective for long-term weight management, even among the general population, and prove especially challenging for the SCI population.^9^ Further, the profound body composition changes noted in chronic SCI coupled with diminished activity and low metabolic demand lead to total daily energy expenditure that is frequently well below daily caloric intake.^10^ The most effective intervention to induce significant and durable weight loss in extremely obese persons is weight-loss surgery (WLS). Specifically, WLS is the only obesity treatment that has been associated with long-term weight maintenance,^11^ long-term remission and prevention of diabetes,^12^ and decreased mortality.^13^

Currently, the two most widely utilized bariatric surgical procedures are Roux-en-Y gastric bypass (RYGB) and vertical sleeve gastrectomy (VSG).^14^ The ease of operation and the lower post-operative morbidity has led to VSG becoming the bariatric surgery of choice. Surprisingly, only a few reports of single-subject WLS in the SCI population are available.^15–17^ Further, surgical intervention that achieves durable results in the able bodied should not be inferred as suitable for the SCI population. Numerous comorbidities inherent to chronic SCI may place persons at greater risk from WLS. For example, healing of decubitus (pressure ulcers) is delayed in SCI patients,^18^ and generalized wound healing is compromised in diabetic patients who may share numerous comorbidities with chronic SCI patients.^19,20^

Pre-clinical models of WLS are established, though none have been applied to animal models of SCI. Although WLS procedures are widely accepted for persons without SCI, the case-report literature for persons with SCI is, in our opinion, rudimentary.^15–17^ Emerging pre-clinical mechanistic studies suggest an acceleration of dysregulated gustatory and homeostatic circuits post-SCI.^21^ However, studies of central nervous system remodeling of energy homeostasis brought about by WLS are a critical knowledge-gap. Using our established model of gastric dysfunction,^22–25^ we tested the principal hypothesis that VSG surgery in T3-SCI rats that had been rapidly rendered obese by consumption of a high-fat diet (HFD) would present minimal complications from WLS; while providing similar weight-loss benefits as those observed in neurally intact control rats. To test the tolerance of WLS in this model, we evaluated the weight loss and overall physical well-being for 6 weeks after VSG surgery.

## Methods

### Animals

Adult, male Wistar rats (*n* = 20; 200–224 g upon receipt; Envigo, Indianapolis, IN) were used exclusively to limit additional hormonal variability. Rats were randomized and remained on a standard laboratory diet (STD; Teklad 2018, 6.2% kcal from fat, 4.00 kcal/g; Envigo) or fed a Western HFD (HFD; 60% kcal from fat, 5.13 kcal/g; Research Diets, New Brunswick, NJ) beginning 1 week before assignment into the experimental groups in order to acclimate to the novel diet. Animals were initially housed 2 per cage on a 12-h light/dark cycle with food and water provided *ad libitum* and were housed singly with environmental enrichment after the first surgical procedure.

### Surgical procedures

All animal usage was in accordance with National Institutes of Health guidelines and was approved by the Institutional Animal Care and Use Committee at the Penn State University College of Medicine. For all surgical procedures, animals were anesthetized with isoflurane (3–5%, 1 L/min O_2_) to achieve areflexia and administered a prophylactic antibiotic (Enroflox, 10 mg/mL; Bayer, Leverkusen, Germany) subcutaneously to reduce post-surgical infection. For pain relief, animals received a single pre-operative dose of sustained release buprenorphine (1 mg/kg; Reckitt Benckiser Pharmaceuticals, Richmond, VA). After exposure of the T3 spinal cord, a 300-kdyne impact (15-sec dwell time) was administered to the T3 spinal segment.^23^ Surgical control animals underwent identical laminectomy and spinal exposure procedures, with the exception of the contusion injury. Post-operative maintenance consisted of daily body weight and food intake, bladder expression, supplemental fluids, and wound care as required, as well as general inspection for complications stemming from limited mobility accompanied by excessive weight gain.

After 7 weeks of STD or HFD feeding (1 week of acclimation +6 weeks after first surgery), all animals were reanesthetized for VSG or a sham surgery.^26^ Briefly, a midline laparotomy was performed to expose the ventral surface of the stomach. Next, the stomach was divided by using an ENDOPATH ETS 35-mm straight endocutter (Ethicon Endo‑Surgery, Inc., Cincinnati, OH). The staple line on the lesser curvature was placed 2–3 mm below the gastroesophageal junction. On the greater curvature, it was placed to form a tubular gastric pouch representing ∼20% of the original stomach size. The abdominal incision was closed, and all rats were injected subcutaneously with normal saline (50 mL/kg). Rats received a clear liquid diet (Boost™, *ad libitum*) for 1 week post-VSG to facilitate healing of the gastric wound margin, after which time they were returned to the pre-assigned diet for an additional 6 weeks. Post-VSG maintenance consisted of continued daily body weight and assigned diet consumption, wound care as required, as well as general inspection for complications (e.g., cellulitis, edema). Tolerance to VSG post-SCI was based upon clinical assessment of wound healing and signs of internal abdominal infection by investigators and Veterinary staff.

At the termination of the study, rats were euthanized and perfused transcardially with 4% paraformaldehyde. The spinal cord in the vicinity of the original laminectomy was extracted for subsequent cryosectioning and staining with luxol fast blue for histological verification of lesion severity.^23^

For each animal, percent sparing of white matter was calculated and analyzed by a *t*-test (Sigmaplot 12.5; SYSTAT, Palo Alto, CA). Food intake was averaged over 1-week periods and normalized as mean energy intake (MEI; defined as kcal•day^−1^•100 g body wt^−1^). The analysis for percent change in MEI was compared between 3 weeks after recovery from a surgical procedure versus 7 weeks after a surgical procedure and was analyzed by two-way analysis of variance and Tukey's *post hoc* tests. MEI for the 2 weeks after a surgical procedure was not analyzed because of potential confounds by post-operative care. Data shown in the figures are mean ± standard error of the mean, and significance was set at *p* < 0.05.

## Results

**FIG. 1.**
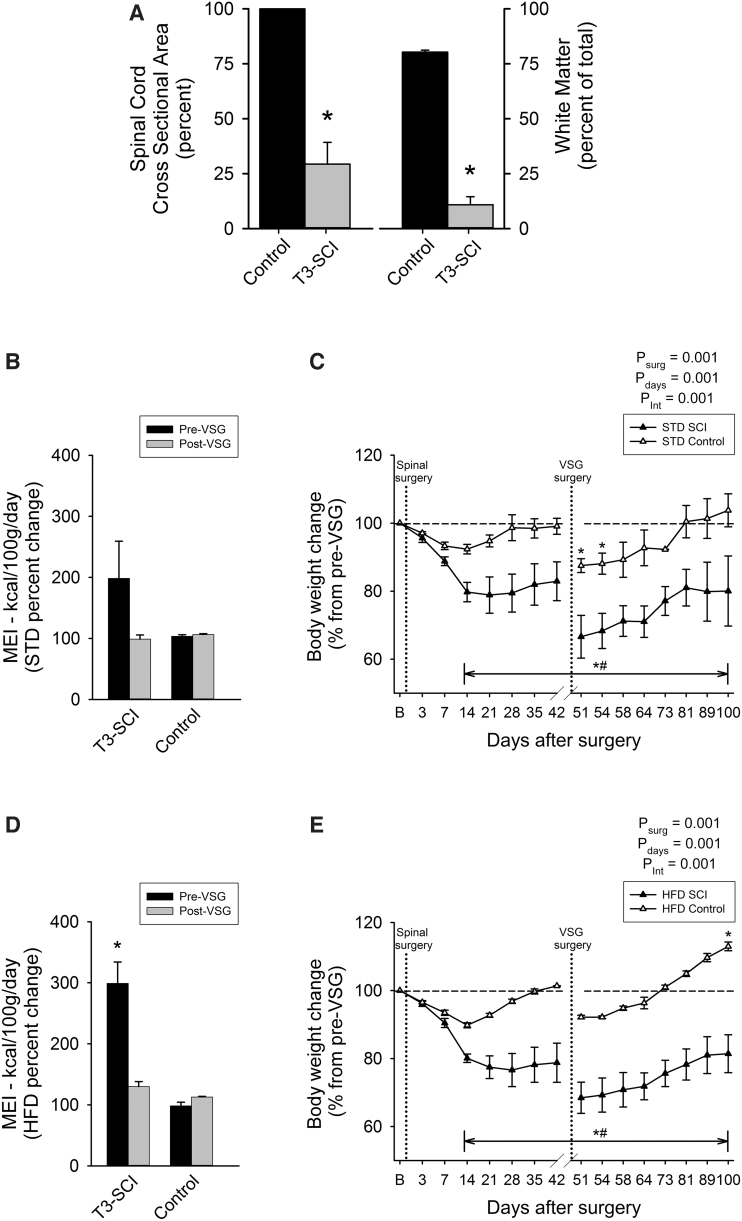
Vertical sleeve gastrectomy (VSG) is equally effective in both T3-SCI and surgical control rats. (**A**) Summary plot of lesion center tissue loss after T3-SCI (percent of cross-section and percent of white matter sparing). No differences in lesion extent were observed between diets. (**B**) Mean energy intake (MEI) beginning after a 2-week recovery period after T3-SCI or control surgery. Over the course of the 4-week monitoring period before vertical sleeve gastrectomy (pre-VSG), there was a trend toward a greater MEI of a standard diet (STD; 6.2% calories from fat, 4.00 kcal/g) in T3-SCI rats compared to surgical controls. After a 2-week recovery period post-VSG, STD MEI of over an additional 4-week monitoring period returned to levels similar to control. (**C**) The VSG procedure significantly reduced body weight in *ad lib* STD diet-fed rats that persisted for 4 weeks after VSG. (**D**) Rats fed a high-fat diet (HFD; 60% calories, 5.13 kcal/g) after T3-SCI demonstrated a significantly greater MEI compared to surgical controls. (**E**) After VSG, body weight change was significantly reduced in both T3-SCI and surgical controls. *p < 0.05 versus pre-operative baseline weight; ^#^*p* < 0.05 versus corresponding surgical controls. SCI, spinal cord injury.

Percent area of white matter at the lesion epicenter of T3-SCI rats was significantly reduced in comparison to T3-control animals (Fig. 1A; *p* < 0.05). There was no significant difference between T3-SCI rats that were fed an HFD compared to those fed an STD, and the lesion center data was pooled. The data for control animals were also pooled in Figure 1A for clarity. These data are comparable to the injury extent reported previously and indicate the severity of our injury model.^24,25,27,28^

For the first stage of the experiment, no surgical control animals experienced post-operative complications. After T3-SCI surgery, 4 rats died because of post-injury complications (Table 1). One case of aspiration reflects an infrequent event that was attributable to impaired gastric emptying post-SCI.^24^ The remaining 2 rats were euthanized by veterinary order because of bladder or circulatory complications.

**Table 1.** Occurrence and Cause of Mortalities for Entire Study

Surgical procedure	Diet group	Days after surgery	Complication
T3-SCI	Standard chow	1 day after SCI	Aspiration of gastric contents attributable to dysmotility
T3-SCI	Standard chow	4 days after SCI	Unknown cause
T3-SCI	High fat	1 day after SCI	Unknown cause
T3-SCI	High fat	5 days after SCI	Unknown cause
T3-SCI	High fat	Euthanized after SCI	Severe edema of feet
T3-SCI	High fat	Euthanized after SCI	Severe bladder obstruction

SCI, spinal cord injury.

No animals that received control spinal surgery and a subsequent VSG bariatric procedure 7 weeks later experienced any adverse consequences immediately after VSG surgery (Table 1). However, 1 surgical control rat died because of unknown causes after an otherwise unremarkable 3-week recovery period. One T3-SCI rat was euthanized by veterinary order because of reopening of the abdominal wound margin 1 day after VSG surgery.

MEI (food intake normalized to body weight) of STD is severely diminished after T3-SCI before stabilizing after 2 weeks (reviewed in a previous work^29^). Analysis of MEI beginning after a 2-week recovery period after T3-SCI or control surgery revealed a non-significant trend toward a greater MEI in T3-SCI rats compared to surgical controls (Fig. 1B; *p* > 0.05). After a 2-week recovery period after the VSG surgery, MEI over the 4-week monitoring period returned to levels similar to control (Fig. 1B). The resulting body weight after the initial spinal surgery was significantly reduced in T3-SCI rats while returning to baseline in surgical controls (Fig. 1C; *p* < 0.05). After a 2-week recovery period after the VSG surgery, body weight decreased significantly further before returning to pre-VSG levels in control and T3-SCI rats. T3-SCI rats remained significantly below the original starting body weight (Fig. 1C; *p* < 0.05).

Rats fed an HFD after T3-SCI demonstrated a significantly greater MEI compared to surgical controls (Fig. 1D; *p* < 0.05). After a 2-week recovery period after VSG, MEI returned to control levels (Fig. 1D; *p* > 0.05). After VSG, body weight was significantly reduced in both T3-SCI and surgical controls. After a 2-week recovery period after the VSG surgery, body weight displayed a non-significant decrease before returning to levels greater than the original value in control rats, whereas T3-SCI rats remained significantly below the original starting body weight (Fig. 1E; *p* < 0.05).

## Discussion

This study investigated the feasibility of performing weight-loss surgery in a pre-clinical model of T3-SCI. Despite initial mortalities after T3-SCI, the VSG bariatric procedure was successfully implemented in a rat model with chronic (≥7 weeks) high-thoracic SCI similar to neurally intact surgical control rats. Further, animals with T3-SCI exhibited a greater reduction in consumption of the highly palatable HFD utilized experimentally to accelerate post-SCI weight gain. Obesity is the net result when energy intake exceeds energy expenditure. Animal studies have been extensively used to model human obesity, and the use of an HFD in this study to accelerate the obesogenic process has long been recognized.^30,31^ Although body composition was not assayed in this study, the post-SCI weight loss by our rats before VSG surgery is considered to be attributable to lean mass reduction (sarcopenia) below the level of injury.^32,33^

Unlike the profound alterations in gastroduodenal anatomy resulting from RYGB, the less invasive VSG procedure is limited to reduction of the stomach to a small sleeve through which ingested foods reach the duodenum. The mechanisms responsible for the positive outcomes of VSG, more so than for RYGB surgery, are not fully understood and are acknowledged to be multi-factorial. The benefits of WLS are not a result of malabsorption or diminished gastric capacity (see a previous work^34^). Instead, increasing evidence suggests that changes in taste, food preferences, and food reward are key contributors to the weight loss observed after RYGB surgery.^27,29^ Specifically, RYGB patients voluntarily restrict consumption of calorie-dense, highly palatable foods.^35^ Similar shifts in preference for fatty and sweet foods after RYGB have been reported in rodents.^36,37^ Regardless of surgical approach, these mechanisms remain to be investigated in the rodent SCI model now that we have proven the feasibility of performing the less complex VSG procedure in animal models with previous chronic SCI.

Gastrointestinal signaling consists of mechano- and chemosensory signals. Both WLS procedures reduce afferent signaling from the mechanoreceptors lining the gastrointestinal mucosa and smooth muscle that detect both tension (stretch) exerted upon the gut wall as well as stimuli regarding composition and density of the chyme.^38^ Additionally, afferent fibers lining the gastric wall detect chemical mediators released by specialized cells lining the lumen of the gastrointestinal tract. For example, the chemical composition of macronutrients as well as other chemicals within the gastrointestinal lumen triggers the release of a wide range of peptides (e.g., cholecystokinin, glucagon-like peptide 1 [GLP-1], and ghrelin) and classical neurotransmitters (e.g., serotonin) from enteroendocrine cells as part of both a paracrine and endocrine signaling cascade.^39,40^ Both RYGB and VSG have been shown to cause significant increases in post-prandial satiety hormones, such as GLP-1 and peptide YY, although the necessity of these changes for weight-loss outcomes have recently been debated.^41^

Further, whereas post-surgical plasma levels of the hunger hormone, ghrelin, produced by the stomach, vary greatly after RYGB, they appear to be consistently reduced after VSG in both clinical and pre-clinical studies.^34,42,43^ These gut-brain hormones are released during feeding or fasting and are detected by vagal sensory terminals in the gut wall as well as on the vagal afferent cell bodies within the nodose ganglia (discussed in a previous work^44^). Accumulating evidence includes altered gastrointestinal peptide signaling,^45^ altered gut microbiota,^46^ plasticity of gut-brain neurocircuitry, and neurohormonal modulation of appetite after WLS.^47^ The apparent reduction of gastrointestinal chemosensory signaling to medullary neural circuits^22,25,44^ raises the question of pathophysiological remodeling of vagal afferent fibers post-SCI and the resulting changes superimposed by excision of the vagally innervated gastric wall as well as possible alterations in reward functions.^34^

One of the limitations of our study is the lack of taste preference data that would indicate whether the gustatory changes observed after bariatric surgery are attributable to alterations in taste and gut-brain nutrient/reward sensing. Such nutrient/reward sensing studies would require larger sample sizes than those used for this feasibility study. In addition, techniques to quantify metabolic parameters with indirect calorimetry and activity monitors are available to investigators as is whole-body composition analysis of animal models to provide ratios of lean, fat, and bone mass. However, it should be noted that determining lipid compartmentalization becomes increasingly difficult once subjects reach a higher fat mass. The potential for WLS to exacerbate sarcopenia, neurogenic bowel, and loss of esophageal tone are also potential avenues for pre-clinical neurophysiological studies.

In conclusion, here we show that WLS in T3-SCI rats is tolerated and provides durable weight loss. Thus, this rodent model is a viable tool for addressing mechanistic questions regarding obesity post-SCI and the development of translational strategies.

## Abbreviations Used

BMI: body mass index
GLP-1: glucagon-like peptide 1
HFD: high-fat diet
MEI: mean energy intake
RYGB: Roux-en-Y gastric bypass
SCI: spinal cord injury
STD: standard laboratory diet
VSG: vertical sleeve gastrectomy
WLS: weight-loss surgery
